# MiniGPT-Pancreas: Multimodal Large Language Model for Pancreas Cancer Observation and Localization in CT Images

**DOI:** 10.1007/s41666-025-00224-6

**Published:** 2025-11-17

**Authors:** Andrea Moglia, Elia Clement Nastasio, Luca Mainardi, Pietro Cerveri

**Affiliations:** 1 Department of Electronics, Information, and Bioengineering, Politecnico di Milano, via Giuseppe Ponzio 34, 20133 Milan, Lombardy Italy; 2 Department of Information Engineering, Università di Brescia, via Branze 38, 25123 Brescia, Lombardy Italy; 3 Department of Industrial, and Information Engineering, University of Pavia, via Adolfo Ferrata 5, 27100 Pavia, Lombardy Italy

**Keywords:** Vision-language models medical imaging, Multimodal large language models medical imaging, Multimodal large language models pancreas, Artificial intelligence pancreas, Generative artificial intelligence medical imaging, AI pancreas

## Abstract

Pancreatic cancer remains one of the deadliest malignancies, primarily because of its subtle CT appearance and frequent late-stage diagnosis. We introduce MiniGPT-Pancreas, a lightweight multimodal large language model (MLLM) that interprets natural-language queries within an interactive ChatGPT-style interface, as well as computed tomography images, and returns precise bounding-box predictions for the pancreas and associated tumors. A cascaded fine-tuning strategy was applied to MiniGPTv2, a multi-task general-purpose MLLM, with a focus on pancreas and tumor detection, using the National Institute of Health (NIH) and Medical Segmentation Decathlon (MSD) pancreas datasets. Pancreas detection achieved an average intersection over Union (IoU) of 0.57 on NIH and MSD datasets, outperforming the base MiniGPT-Pancreas model and more recent MLLMs like GLM-4.1V-9B-Base (general-purpose) and UMIT (specific to the biomedical domain). Tumor observation on MSD yielded an accuracy, precision, recall, and F1 score of all about 0.87, surpassing MiniGPT-v2, GLM-4.1V-9B-Base, and UMIT. For tumor localization, the IoU was 0.28, higher than UMIT (IoU=0.07), but lower than GLM-4.1V-9B-Base (IoU=0.48). On multi-organ detection on the AbdomenCT-1k dataset, MiniGPT-Pancreas outperformed GLM-4.1V-9B-Base and UMIT in all organs, with an IoU of 0.50 on pancreas vs. 0.43 and 0.03, respectively. MiniGPT-Pancreas was rated highly by an international group of 10 expert general surgeons (Italy, Singapore, and the UK) as a potential training tool, especially for verification (4.5/5.0), and training of young specialists (4.5/5.0) on a 5-point Likert scale. While operating on 2D slices limits volumetric context, MiniGPT-Pancreas demonstrates that compact MLLMs can rival specialized vision networks in pancreas imaging, offering an intuitive, language-driven tool for AI-assisted radiology. The code is publicly available at https://github.com/elianastasio/MiniGPTPancreas.

## Introduction

Pancreatic cancer is notoriously deadly, with a five-year survival rate of 13% in the United States, making it the lowest one among all cancer types [1]. Its lethality is compounded by the fact that surgical resection is the only potentially curative treatment, but only a small fraction of patients are eligible for surgery due to the advanced stage at diagnosis time. Even among those who undergo surgery, recurrence rates are high, and long-term survival remains low. For this reason, an early diagnosis of pancreatic cancer is crucial. Diagnosis involves clinical assessment, laboratory testing, and advanced imaging techniques like computed tomography (CT). Analysis of pancreas radiological imaging is demanding for several reasons. First, the pancreas has an irregular shape and can be easily deformed. Its shape, size, aspect ratio, position, and orientation inside the abdominal cavity vary largely among subjects [2, 3]. Second, the organ is very small, and its volume is generally less than 0.5% of the entire CT [3]. Third, there is low contrast in the CT slices between the pancreas boundaries and the other abdominal structures. Unfortunately, tumors smaller than 2 cm often evade detection by CT, emphasizing the need for new methods to support clinicians [4]. Artificial intelligence (AI) models have made great strides in screening, diagnosis, treatment guidance, and prognosis prediction in oncology over the past few years [5]. However, AI models face challenges in pancreatic cancer imaging as traditional approaches [6]. For instance, state-of-the-art AI architectures hardly reach an accuracy of 0.60 on tumor segmentation on commonly public datasets. This is lower compared to cancers in other organs, such as the liver, where an accuracy of up to 0.70 has been reported [7]. More recently, large language models (LLMs), a type of AI model pre-trained on massive data using unsupervised learning and fine-tuned on specific downstream tasks, were applied to healthcare. They have demonstrated their capability to pass medical examinations, requested by national boards in different countries to obtain the license for clinical practice [8]. However, those LLMs are unimodal. As such, they are not suitable for patient diagnosis and treatment, which mandates a holistic approach combining different sources of data, e.g., medical history, electronic health records, laboratory tests, and radiology results [9]. By combining multimodal data, e.g., text and image, multimodal large language models (MLLMs) have the potential to revolutionize healthcare, for instance, by interpreting patient history or summarizing findings. Moreover, the ability of LLMs, and consequently MLLMs, to process natural language queries and provide straightforward answers could streamline clinical decision-making processes. Furthermore, the capabilities of MLLM-based chatbots could prove instrumental in assisting young medical practitioners in scenarios when they need a second opinion from a more experienced colleague. Thus, MLLM-based medical chatbots may relieve the burden on the healthcare system, thus improving efficiency [10].

LLaVA-Med, Med-Flamingo, and Med-PaLM M represented initial instances of generalist MLLMs accepting text and images from different modalities, e.g., CT, X-ray, and Magnetic Resonance Imaging (MRI), and generating text as output for tasks in radiology, dermatology, and pathology [11–13]. As such, they were specialized for the visual question answering (VQA) task, designed to answer an image-related question according to the image content, for instance, to formulate a diagnosis based on a radiological image and patient data [10]. However, a disease diagnosis should not be limited to VQA, but based on a holistic approach to provide clinicians with key information, e.g., the localization of the disease, its extension, and even its classification. To pursue this ambitious goal, it is therefore crucial that the AI models be equipped with broader reasoning capabilities for medical diagnosis. Unfortunately, developing and training an MLLM for the above-mentioned tasks in medical diagnosis is challenging for several reasons. First, in medicine, there is no availability of massive data for pre-training of the same order of magnitude as for general-purpose models. Second, curating a large-scale dataset for medical applications requires expert annotation, which is expensive in terms of time and cost. Third, collecting patients’ data, e.g., medical images, requires privacy protection to comply with security laws, like the European Union General Data Protection Regulation (GDPR). Fourth, even if these datasets existed, the cost of the necessary hardware for pre-training and fine-tuning an MLLM would be prohibitive for most research centers, especially academic ones. Therefore, a possible solution could be to fine-tune an existing MLLM for specific tasks. MiniGPT-v2 was proposed as a versatile general-purpose MLLM not limited to image captioning and VQA tasks as LLaVA-Med, Med-Flamingo, and MedPaLM M, but capable of performing additional tasks, such as referring expression comprehension (REC), i.e., identification and detection of a specific object in an image that is referred to by a natural language expression [14]. MiniGPT-Med, a fine-tuned example of MiniGPT-v2, was developed for several tasks related to lung diagnosis, e.g., detection of pneumonia [15]. However, at present, there is no published study on MLLMs addressing the challenges of pancreatic cancer, e.g., localizing a tumor inside a CT scan after receiving a text prompt. To tackle this issue, we propose MiniGPT-Pancreas, an MLLM for pancreas detection, tumor detection, and classification tasks from CT scans. It is based on a cascade fine-tuning of MiniGPT-v2 on the National Institute of Health (NIH), and the Medical Segmentation Decathlon (MSD), two publicly available datasets [16, 17]. It was finally tested on the AbdomenCT-1k dataset for the multi-organ detection task of the abdomen [18]. The main contributions of this work are the following:

- we introduced MiniGPT-Pancreas, the first MLLM for pancreas diagnostic support, allowing interactive dialogue with users in a conversational ChatGPT style;
- we performed a cascade fine-tuning and extensive evaluation of MiniGPT-Pancreas for pancreas detection, tumor observation, and finally, tumor localization. More specifically, for each new task, MiniGPT-Pancreas was fine-tuned on the checkpoint of the previous task;
- we showed that MiniGPT-Pancreas achieved superior performance to MiniGPT-v2 by a large margin in all the tasks, including multi-organ detection on the AbdomenCT-1k dataset with annotations of the kidney, liver, pancreas, and spleen. It outperformed more recent MLLMs in almost all the tasks.

The remaining of this manuscript is structured as follows: in Section 2 we report the related work on MLLM for medicine. In Section 3, we describe the architecture of MiniGPT-Pancreas, the prompt template, the preprocessing of CT volumes, the training pipeline, the evaluation metrics, and the implementation of the training sessions. In Section 4, we report the results on pancreas detection, tumor classification, and the detection task; we also present the results on the multi-organ detection task. In Section 5, we discuss our findings, along with limitations and future developments. Section 6 ends the manuscript.

## Related Work

Traditional medical VQA tasks were approached by AI models as classification problems, where the model is trained to categorize image-text representations into a predefined set of answers. However, this approach was limited by its reliance on a fixed set of candidate answers, restricting its utility for open-ended questions that are common in medical diagnostics [13]. Recent advancements have led to the development of several MLLMs generating open-ended responses rather than selecting from pre-defined answer choices in the healthcare domain [13].

BiomedGPT was the first open-source MLLM for healthcare, capable of performing VQA, report generation, and summarization [19]. It was pre-trained on 14 freely available datasets and fine-tuned on 19 datasets for VQA and image captioning tasks. Its architecture consisted of an encoder-decoder LLM and a convolutional neural network as the visual encoder. BiomedGPT outperformed the state-of-the-art (SOTA) models on the image classification task [19].

Med-PaLM M was proposed by Google (Mountain View, CA, United States) for radiological image classification, VQA, report generation, and summarization [20]. It was based on the Pathways Language Model (PaLM) decoder transformer and a vision transformer as the visual encoder. Med-PaLM M was developed by fine-tuning PaLM-E on 12 open-source datasets and 14 tasks. It scored better than SOTA approaches on VQA, report generation, and image classification [20].

XrayGPT was designed as a conversational MLLM for analyzing chest radiographs and generating summaries from radiological reports [21]. It consisted of a visual encoder, Vicuna as LLM, and a linear projection layer to align visual features with the LLM. During training, only the linear transformation layer was updated. XrayGPT leveraged the visual encoder of MedCLIP, whose design was inspired by Contrastive Language–Image Pre-training (CLIP), with the distinction of decoupling image and text for contrastive learning, enabling the use of text-only and image-only data in the medical field [22, 23]. It was trained in two stages using MIMIC-CRX and OpenI datasets, both containing X-ray images and reports [24, 25].

PMC-CLIP was introduced as an adaptation of CLIP, pre-trained on ROCO, MedICaT, and MIMIC-CXR datasets, and fine-tuned on PMC-OA, a biomedical dataset of 1.6 million image-captions pairs from PubMed. PMC-CLIP outperformed the SOTA, represented by M^3^AE (multi-modal masked autoencoder for medical vision-and-language pre-training), on the VQA-RAD dataset [26, 27].

BiomedCLIP was designed as an adaptation of CLIP to the biomedical domain, trained on PMC-15M, a dataset consisting of 15 million biomedical image-text pairs from PubMed. Its architecture was based on the PubMedBERT text encoder instead of GPT-2 as the original CLIP, and a larger vision transformer for images with higher resolution [28]. BiomedCLIP outperformed PubMedCLIP on the VQ-RAD and SLAKE datasets for VQA [28].

LLaVA-Med, an extension of LLaVA (Large Language and Vision Assistant), was specifically designed for the medical domain [11]. It was fine-tuned on PMC-15M, and filtered using GPT-4. The development of LLaVA-Med followed a two-stage learning process. In the first stage, the model was trained using image-text pairs to predict the original image caption to ensure that the visual and textual concepts in the biomedical domain were accurately aligned. The second stage involved instruction-tuning, where the model learned to follow more complex and open-ended medical instructions. One of the key innovations in LLaVA-Med was the novel data generation pipeline, leveraging GPT-4 to create diverse instruction-following instances from the PMC-15M dataset without the need for manual annotation. This approach ensured that the model was trained on a wide range of medical concepts, improving its zero-shot performance (the ability of a model to perform a task without having been explicitly trained on any examples of that task) for medical VQA. Experiments showed that LLaVA-Med outperformed its general-domain model, LLaVA, in various benchmark datasets such as VQA-RAD, Slake, and PathVQA, often achieving state-of-the-art results[29, 30].

Medical Visual Instruction Tuning (MedVInT) was designed with CLIP and LLaMA as, respectively, visual and language backbone [31]. MedVInT was pre-trained on PMC-VQA, a dataset of 227k VQA samples from different image modalities and diseases. It outperformed M^3^AE, and PMC-CLIP on both VQA-RAD and SLAKE datasets for VQA, and PMC-CLIP, Open-Flamingo, and LLaVA-Med PMC-VQA dataset [31].

Med-Flamingo, a vision-language model based on OpenFlamingo-9B for the medical domain, was developed to handle complex, interleaved image-text data for few-shot learning (the ability of an MLLM/LLM to make a prediction after receiving a prompt with few examples) [12, 32]. Med-Flamingo was pre-trained on interleaved image and text data from medical textbooks, and PMC-OA as paired data. It overcame the inability to perform in-context learning with few-shot examples of previous medical MLLMs, such as ChexZero, BiomedCLIP, and MedVInT [28, 33].

PeFoMed (Parameter Efficient Fine-tuning of Multimodal Large Language Models for Medical Imaging) was designed by fine-tuning MiniGPT-v2 using low-rank adaptation (LoRA) to reduce computational resources [13, 34]. PeFoMed fine-tuning process involved a two-stage approach, keeping the vision encoder and language model frozen, while updating only the linear projection component and the LoRA layers in both stages. In the first one, the model was fine-tuned using ROCO, a dataset coupling medical images and their corresponding textual descriptions to adapt the model to understand and generate medical text based on visual inputs [35]. In the second stage, the model was fine-tuned on the VQA-RAD dataset [29]. Remarkably, PeFoMed outperformed GPT-4 by a significant margin on the VQA-RAD dataset, particularly for open-ended questions. PeFoMed outperformed LLaVA-Med on the VQA task on the VQA-RAD dataset on both open-ended and closed-ended questions, achieving an overall score of 81.9% of correct answers vs. 75.8% [13].

CheXagent, an MLLM specifically focused on chest X-rays, was trained in four stages on 28 public datasets for eight tasks like VQA, disease classification, and abnormality detection [36]. It was based on Mistral-7B-v01 LLM and BLIP-2 visual encoder [37]. In the first training stage, CheXagent was trained with different sources like PubMed Central abstracts and medical terms from Wikipedia. In the second one, BLIP-2 was trained for image-text contrastive learning and image captioning objectives using image-text pair datasets such as MIMIC-CXR. In the third one, the model was trained using the image captioning objective to bridge the LLM and the visual encoder. In the last stage, CheXagent was trained on a variety of tasks. CheXagent outperformed XrayGPT, Med-Flamingo, RadFM, and LLaVA-Med on several tasks, including VQA, and disease classification [36].

Radiology Foundation Model (RadFM) was developed starting from pre-training on the Medical Multi-modal Dataset (MedMD), consisting of 16 million 2D and 3D images collected from different datasets and accompanied with textual descriptions, such as radiology reports, and visual-language instructions [38]. It was then fine-tuned on three million multimodal samples of MedMD with only radiologic images. RadFM consisted of a 3D visual transformer and MedLLaMA-13B, a fine-tuned version of LLaMA-13B. RadFM outperformed MedFlamingo and GPT-4V on VQA, report generation, and rationale diagnosis tasks [38].

UMIT was designed to handle different tasks like VQA, report generation, image classification, disease detection, and landmark detection, on 2D and 3D images from X-ray, CT, and positron emission tomography (PET) [39]. It was trained on several datasets using a two-stage strategy, namely the feature alignment stage to align visual and textual features, and the fine-tuning stage with instructions, similar to LLaVA-Med to share knowledge across multiple tasks. UMIT outperformed SOTA models on five tasks [39].

**Fig. 1:**
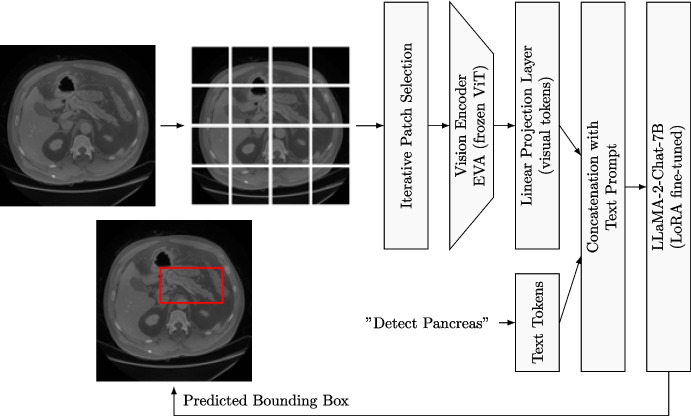
Architecture of MiniGPT-Pancreas consisting of the EVA vision encoder, one linear projection layer, and an LLM. The visual encoder is fed with each CT slice and one text prompt, e.g., a question. The extracted features by the vision encoder are converted by the projector into the input embedding requested by the LLM. They are concatenated with the text token. MiniGPT-Pancreas outputs the coordinates of the bounding box in text format. This is finally drawn as a rectangular bounding box overlaid on the original image. Adapted from [15, 40]

## Materials and Methods

### Model Architecture

MiniGPT-Pancreas leveraged the architecture of MiniGPT-v2 (Fig. 1). It combined a visual backbone, a projection layer, and a pre-trained LLM [14]. As LLaVA-Med, MiniGPT-v2 belongs to the category of auto-regressive multimodal LLMs, which tokenize 2D images (e.g., CT slices) into visual tokens, converted by the projector into the embedding shape requested by the LLM [40]. The converted visual tokens were concatenated with text tokens, both sent as input to the LLM (Fig. 1). EVA, a vanilla vision transformer (ViT) with 1 billion parameters, served as the visual backbone of MiniGPT-Pancreas for image encoding [41]. EVA was fed with $224 \times 224$ pixels images, subdivided into 256 patches, each of $14\times 14$ pixels. The output of EVA was a $1408\times 256$ vector with a hidden size of 1408. To reduce the computational cost, four adjacent visual tokens were fused to one, making the output of EVA equal to $1408\times 64$. A linear projection layer was placed between the visual encoder and the LLM to serve as a bridge between the visual and textual modalities. Its role was to translate the extracted features from the EVA into input tokens compatible with the LLM format. Since the hidden size of LLaMA-2-chat-7b-hf is 4096, the projection layer converted the output of EVA from $1408\times 64$ to $4096\times 64$. By aligning the dimensionality of the ViT output with that of the language tokens, the projection layer ensured that the LLM could process the visual information in a manner consistent with textual data. This transformation was crucial for the seamless integration of visual and textual inputs, allowing the LLM to interpret and generate contextually appropriate responses based on the visual cues provided by the ViT. LLaMA-2-chat-7b-hf (Table 1), a seven-billion-parameter model belonging to the open-source LLaMA-2 ecosystem, was employed as LLM [42]. As can be seen in Table 1, apart from the input and output components, responsible for embedding the text and visual prompts into tokens, the model consisted of 31 layers, formed by a multi-layer perceptron (MLP) and a self-attention component. The rotary embedding inverse frequency is a particular positional embedding technique applied within the self-attention mechanism. It was used to encode positional information into the model more effectively, especially for long sequences. By rotating the query and key vectors in the attention mechanism based on their position within the sequence, and modulating these rotations with an inverse frequency factor, the model can better preserve the relative positional relationships between tokens. This method allowed the model to handle various scales of positional information, ensuring that it captured both short-term and long-term dependencies within the input sequence efficiently. The chat suffix refers to the additional fine-tuning process that the LLM underwent, optimizing it for dialogue use cases. The hf suffix stands for human feedback and refers to reinforcement learning from human feedback, a technique where humans evaluate the model outputs, e.g., responses in a conversation, and provide feedback on their quality. This feedback adjusted the model’s behavior, making it more aligned with human preferences. During the training of MiniGPT-v2, the visual encoder was kept frozen, while the projection layer and the LLM were updated [14].

**Table 1:** Architecture overview of LLaMA-2, all weights are in half precision (F16), meaning that each weight occupies 16 bits in memory. In total, the model accounts for 7 billion parameters. Each of the 31 layers is composed of a self-attention component and an MLP component

Component	Shape
Embed Tokens	[32 000, 4 096]
Layer x 31	
- Input LayerNorm	[4 096]
- MLP Down Projection	[4 096, 11 008]
- MLP Gate Projection	[11 008, 4 096]
- MLP Up Projection	[11 008, 4 096]
- Post Attention LayerNorm	[4 096]
- Self-Att. K Projection	[4 096, 4 096]
- Self-Att. Q Projection	[4 096, 4 096]
- Self-Att. V Projection	[4 096, 4 096]
- Self-Att. O Projection	[4 096, 4 096]
- Self-Att. Rotary Embedding Inverse Frequency	[64]
Norm Weight	[4 096]
LM Head Weight	[32 000, 4 096]

### Prompt Template

MiniGPT-v2 introduced a distinctive feature: a multitask instruction template that inserted a task-specific token to generate the most appropriate output format for each task, e.g., vqa for VQA and refer for REC tasks, respectively. The instruction template had the following format:

*[INST]* < *Img* > < *ImageFeature* > <*/Img*> *[Task Identifier] Instruction [/INST]*

The template was composed of three components: the first consisted of visual features extracted by the ViT, the second was the task identifier token, and the third was the instruction input expressed in natural language Without the task identifier, the MLLM could fail to identify the current instruction. For instance, when the model was asked to detect the pancreas in a CT slice, it could generate an answer in natural language (e.g., "between the stomach and the spine") instead of outputting a bounding box as expected. The instruction component represented the natural language part of the prompt (e.g., ’Where is the pancreas?’). During training, an instruction was randomly selected from a list of available candidates, helping the model to generalize better and to reduce the risk of overfitting to a specific text sequence. The following candidate instructions, preceded by the refer task identifier, were designed for the pancreas detection task:

- "[refer] Give me the location of the pancreas"
- "[refer] Give me the position of the pancreas"
- "[refer] Where is the pancreas?"
- "[refer] Where is the pancreas located in this image?"
- "[refer] Which is the position of the pancreas?"
- "[refer] From this image, tell me the location of the pancreas"
- "[refer] Could you tell me the location of the pancreas?"
- "[refer] Where can I locate the pancreas?"

Similar instructions were designed for pancreas tumor detection. For the tumor observation task, the following YES/NO questions were designed (preceded by the vqa task identifier):

- "[vqa] Does the pancreas in the image present a tumor?"
- "[vqa] Is there a tumor in the pancreas shown in the image?"
- "[vqa] Can you see a tumor in the pancreas in this picture?"
- "[vqa] Does the image show a tumor in the pancreas?"
- "[vqa] Is a pancreatic tumor visible in the image?"
- "[vqa] Is the pancreas in this image showing signs of a tumor?"
- "[vqa] Is the pancreas in the image affected by a tumor?"
- "[vqa] Does the pancreas in the picture have a tumor?"

### Bounding Boxes Representation

For grounding tasks based on the localization of objects, like detection, the model represented the spatial location of the bounding boxes coordinates in natural language:

$$\begin{aligned} {< X_{\text {left}}>< Y_{\text {top}}>< X_{\text {right}}>< Y_{\text {bottom}}>} \end{aligned}$$

Each coordinate is an integer normalized on the [0, 100] range. Specifically, $< X_{\text {left}}>< Y_{\text {top}}>$ represents the top-left corner, while $< X_{\text {right}}>< Y_{\text {bottom}}>$ the bottom-right corner.

### Datasets

The NIH dataset included 82 CTs in the arterial phase, with a 512 $\times$ 512 resolution, a number of slices ranging from 181 to 466, and a [1.5-2.5] slice thickness [16]. The pancreas was manually labeled by a medical student and then verified by an experienced radiologist [16]. The images of the MSD dataset were provided by the Memorial Sloan Kettering Cancer Center (New York, NY, United States). Of the 420 CT scans that were acquired in the venous phase, 281 included annotations of pancreas and tumors [17]. The AbdomenCT-1k is a multi-organ dataset with 1,112 CTs with annotations of liver, pancreas, kidneys, and spleen [18]. A cohort of 15 junior annotators (one to five years of experience) used ITK-SNAP to manually segment the organs under the supervision of two board-certified radiologists. Then, one senior radiologist with more than 10 years of experience checked the annotations. After annotation, U-Net models were trained to find the possible errors, which were double-checked by the senior radiologist [18]. The AbdomenCT-1k dataset includes, among the public datasets, the MSD and the NIH ones, where the annotations of liver, spleen, and kidneys were added to those of the pancreas [18].

### Data Preprocessing

**Fig. 2:**
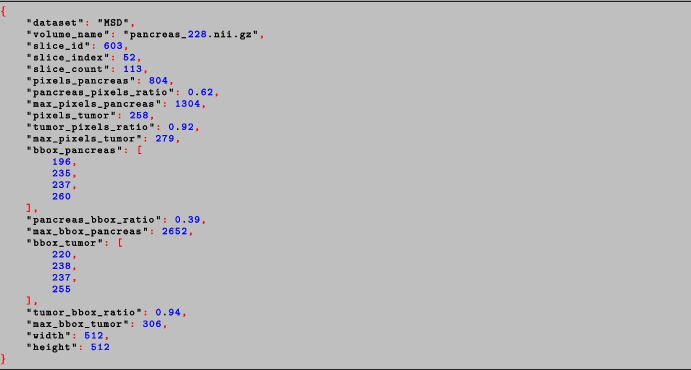
JSON data for a specific pancreas slice from the MSD dataset

In order to use the NIH, MSD, and AbdomenCT-1k datasets during training and testing, some preprocessing steps were performed to convert the 3D CT volumes into 2D images to align them with the input dimensional requirements of the visual encoder of MiniGPT-Pancreas. Additionally, before converting each slice to a PNG file, histogram cropping of 2% and histogram equalization were performed to remove outliers and improve image contrast, respectively. Concurrently with image preprocessing, a JSON file was generated, containing useful annotations for selecting the appropriate slices in the different training processes. An example is shown in Fig. 2. In order to extract the bounding boxes of the anatomical regions of interest (either the pancreas parenchyma or the tumor), the top-left and bottom-right pixels in the annotated mask of the ground truth (GT) were computed. The definitions of all keys used in the JSON file are reported in Section A of the Appendix. Overall, the MSD and NIH datasets included 8,792 and 6,882 slices, respectively. In particular, in each slice, at least one pixel was labeled as pancreas.

### Training Pipeline

The following three training stages were performed:

1. **Pancreas Detection:** During this step, the base checkpoint of MiniGPT-v2 was fine-tuned, yielding MiniGPT-Pancreas. In particular, the model was asked to generate a bounding box around the pancreas, using the [refer] task identifier. Both the MSD and NIH datasets were employed. In order to evaluate the impact of using small or large GT bounding boxes containing the pancreas, 10 different training sessions were performed by choosing a different pancreas_bbox_ratio threshold (equivalent to the ratio between the pancreas bounding box and the largest pancreas bounding box in any slice of the same volume). The threshold ranged from 0% to 90%. This ensured that only the slices with at least that pancreas bounding box area ratio were selected. A train-test split of 80%-20% was chosen, based on the number of CT volumes.
2. **Tumor Observation:** For this task, the MiniGPT-Pancreas model obtained from the previous task was fine-tuned to determine whether the images contained a pancreas tumor or not. In this case, the task identifier [vqa] was selected.
3. **Tumor Localization:** For this task, the fine-tuned MiniGPT-Pancreas version for tumor observation was further fine-tuned to localize the pancreas tumors. The same [refer] task identifier as in the first stage was employed. In this case, only the MSD dataset was used since it contained annotations of tumors, in contrast with the NIH dataset, which included only healthy pancreas. A key difference from the first training stage was the significantly smaller size of the GT bounding boxes.

We compared the performance of MiniGPT-Pancreas against GLM-4.1-9B-Base, a recently introduced general-purpose MLLM [43]. Specifically, GLM-4.1-9B-Base underwent the same fine-tuning pipeline as MiniGPT-Pancreas. Then, we compared the obtained fine-tuned version of these two general-purpose MLLMs with UMIT, an MLLM pre-trained on several large datasets of medical images, one of which was M3D-Seg, a collection of 25 public datasets with CT volumes, including NIH, and MSD-Pancreas [39, 44]. In this case, the comparison was evaluated on the test set.

### Evaluation Metrics

Despite Intersection over Union (IoU) being a standard metric in segmentation tasks, it has a large sensitivity to size, especially with small masks that can reveal dramatic shifts in IoU with minute changes [45]. In addition, IoU does inform where the mismatches were occurring, for instance, whether errors are concentrated along the boundary or spread throughout the mark region. The pancreas and its associated tumors are typically small, irregular, and low-contrast structures in abdominal CT scans. In such scenarios, IoU becomes highly sensitive to small mismatches in boundary delineation, often penalizing otherwise clinically acceptable detections. Recent literature proposed variants of IoU like generalized IoU (GIoU), distance-IoU (DIoU), or complete IoU (CIoU). However, they were signaling low scores even when the organ was present. In particular, IoU variants still penalize boundary differences heavily, even if both masks contain the full organ. Also, if the predicted mask is slightly shifted or misaligned, even with complete coverage, the overlap is low. For example, even a slight spatial shift in a predicted pancreas bounding box can drastically reduce the IoU variants, despite the model correctly identifying the region of interest. Thus, along with the IoU score, we adopted two additional indices, namely mask centroid distance (CD) and organ/lesion inclusion (OI), reflecting positional accuracy and coverage reliability, respectively. Mask CD measured the distance between the label and predicted mask centroids, scaled to the diagonal of the input image size, to the vision encoder as:

1 $$\begin{aligned} \text {CD} = 100 \times \frac{\sqrt{(x_p - x_g)^2 + (y_p - y_g)^2}}{\sqrt{W^2 + H^2}} \end{aligned}$$

where $(x_p,y_p)$, $(x_g,y_g)$ are the centroid coordinates of the predicted mask and ground truth masks. *W*, *H* are width and height of the input image (100$\times$100), respectively. Organ inclusion was set to 1 if the centroid distance was less than half the diagonal of the label mask, 0 otherwise, as:

2 $$\begin{aligned} \text {OI} = {\left\{ \begin{array}{ll} 1, & \text {if } d_c < \dfrac{1}{2} \sqrt{w^2 + h^2} \\ 0, & \text {otherwise} \end{array}\right. } \end{aligned}$$

where $d_c$, *w*, and *h* are the centroid distance between the predicted and ground truth bounding boxes, the width and height of the ground truth bounding box, respectively. Summarizing, CD captures the positional accuracy of predictions, reflecting whether the model is "looking in the right place." OI measures whether the predicted bounding box sufficiently covers the true region, regardless of precise boundary overlap. Together, these metrics offered a spatially interpretable and clinically aligned complement to IoU. In order to complement IoU, CD, and OI metrics, normalized center-point heatmaps were computed across all the test sets for tumor localization. These showed at a glance whether predicted and true bounding-box center clusters were consistent, even for small tumors, revealing any systematic spatial biases. The procedure involved the following: (1) extraction of each box’s centroid, (2) normalization to the image coordinate frame, and (3) aggregation into a 2D density map (see Appendix B).

### Implementation Details

In our experiments, we used all 82 CTs of the NIH, 281 CTs of the MSD-Pancreas, and 1,112 CTs of the AbdomenCT-1k datasets. Data were split into 80%-20% for training and testing, respectively, in each dataset for each task. All training sessions were performed on a single NVIDIA A100 GPU with 40GB of memory. The model was initialized using the checkpoint of MiniGPT-v2. All weights of the ViT were kept frozen, while the linear projection layer was updated. The LLM was trained by applying LoRA to the key and query weights matrices of the attention layers, $W_q$ and $W_k$, with rank set to 64 and scaling factor $\alpha$ set to 16. This allowed us to reduce the number of trainable parameters of the LLaMA-2-chat-7b-hf to just 34 million, equivalent to approximately 0.5% of the original model. Image resolution was set to 448$\times$448 in both training and testing. AdamW was chosen as optimizer, in combination with a linear warm-up cosine scheduler. The initial learning rate was $10^{-5}$, while the warm-up and the minimum learning rate were both set to $10^{-6}$. Weight decay was set to 0.05. Cross-entropy was used as a loss function. Each fine-tuning process ran for 50 epochs, taking approximately 12 hours. Once trained for the respective task, MiniGPT-Pancreas could be used for inference by submitting multimodal prompts to the native ChatGPT-style web interface of MiniGPT-v2, designed with Gradio^1^.

### Questionnaire with Expert Surgeons

A 5-point Likert scale questionnaire, where 1 represented the minimum score and 5 the maximum, was distributed to a panel of ten experts in pancreatic surgery, encompassing specialists in open, laparoscopic, and robotic procedures, to assess the potential of MiniGPT-Pancreas. The survey consisted of 10 targeted questions aimed at evaluating MiniGPT-Pancreas across three key domains: the clinical need it addresses, its integration into the existing clinical workflow, and its possible application as a training tool for the education of clinicians.

## Results

### Pancreas Detection

The number of slices in the train and test splits of the NIH and MSD datasets varied according to the increasing pancreas_bbox_ratio threshold from 0% to 90% (Table 2). Each training session lasted 10 epochs.

**Table 2:** Number of slices in the train and test splits of the NIH and MSD datasets for the pancreas detection task among different thresholds

Threshold (%)	NIH		MSD	
	Train	Test	Train	Test
0	5,520	1,352	6,936	1,803
10	4,066	1,064	5,856	1,526
20	2,705	720	4,391	1,149
30	2,180	614	3,327	893
40	1,891	544	2,710	716
50	1,657	455	2,312	621
60	1,402	378	1,985	526
70	1,180	306	1,681	444
80	897	226	1,389	343
90	504	135	968	224

**Fig. 3:**
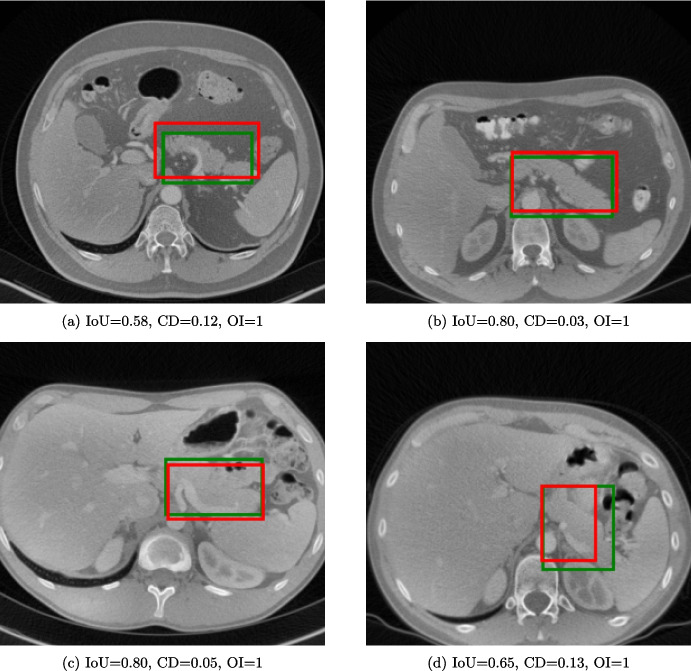
Four slices in the NIH dataset used for the pancreas detection task. The green bounding box represents the GT, while the red represents the prediction

As expected, the IoU scores, computed both on the NIH and MSD datasets, increased with the threshold increase (Table 3). A 60% value of threshold was adopted as a reasonable trade-off between the IoU scores and the number of slices in the train and test split for each threshold level. Using a 60% threshold, results of the model trained for 50 epochs yielded the highest IoU after 33 epochs on the NIH (0.60) and 21 epochs on the MSD dataset (0.55), respectively (Table 4). These values were higher than those obtained by the base MiniGPT-v2 before fine-tuning, i.e., 0.053, 0.043, and 0.047 on the NIH, MSD, and the average between the two datasets, respectively. MiniGPT-Pancreas outperformed GLM-4.1V-9B-Base and UMIT on the average score between the two datasets. With a balanced dataset, the performance of MiniGPT-Pancreas improved, especially on the MSD dataset (Table 5). Since IoU does not account for false positives, accuracy, precision, and F1 score were computed (threshold of 0.5), reporting 0.875, 0.849, 0.862 on the NIH, and 0.890, 0.838, 0.863 on the MSD dataset respectively. The optimally trained model was used to compute centroid distance and organ inclusion, achieving 0.11 and 0.98 for NIH, 0.13 and 0.93 for MSD (Table 6), confirming higher performances in pancreas detection on the NIH dataset. As an example, four cases, displayed in (Fig. 3), reported varying IoU, while CD and OI were more consistent in between.

**Table 3:** Average IoU for the pancreas detection task

Threshold (%)	NIH	MSD	Average
0	0.396	0.408	0.403
10	0.434	0.450	0.444
20	0.469	0.461	0.464
30	0.503	.0474	0.486
40	0.529	0.500	0.512
50	0.550	0.510	0.527
60	0.587	0.541	0.560
70	0.606	0.577	0.589
80	0.626	0.591	0.605
90	0.634	0.599	0.612

**Table 4:** Scores on IoU for the pancreas detection task with the threshold set at 60%. The average was weighted by considering the number of slices. The best results are in bold

	NIH	MSD	Average
Epoch	IoU	IoU	IoU
1	0.574	0.533	0.550
3	0.584	0.544	0.560
5	0.591	0.538	0.560
7	0.587	0.548	0.564
9	0.584	0.548	0.563
11	0.585	0.543	0.561
13	0.592	0.555	0.570
15	0.587	0.552	0.566
17	0.594	0.558	0.573
19	0.593	0.556	0.572
21	0.595	**0.550**	**0.574**
23	0.593	0.554	0.570
25	0.590	0.553	0.569
27	0.592	0.556	0.571
29	0.594	0.555	0.571
31	0.589	0.555	0.569
33	**0.597**	0.556	0.573
35	0.591	0.556	0.571
37	0.589	0.559	0.572
39	0.594	0.557	0.572
41	0.596	0.556	0.573
43	0.595	0.555	0.572
45	0.595	0.556	0.572
47	0.593	0.557	0.572
49	0.594	0.556	0.572

**Table 5:** Comparison between MiniGPT-Pancreas and other MLLMs on pancreas detection, using IoU as evaluation metric. Highest scores are in bold

	NIH	MSD	Average
MiniGPT-v2	0.053	0.043	0.047
GLM-4.1V-9B-Base	0.590	0.566	0.570
UMIT^*^	0.069	0.048	0.059
MiniGPT-Pancreas	**0.597**	0.550	0.574
MiniGPT-Pancreas (balanced)^**^	0.590	**0.628**	**0.609**

^*^Only inference. ^**^Only for true positive cases

**Table 6:** Comparison of Intersection over union, centroid distance, and organ inclusion check metrics (mean ± SD) for pancreas detection

NIH			MSD		
IoU	CD	OI	IoU	CD	OI
0.60(0.17)	0.11(0.06)	0.98(0.11)	0.55 (0.19)	0.13(0.10)	0.93(0.25)

### Tumor Observation and Localization

For the pancreas tumor observation task, the best MiniGPT-Pancreas model, trained on 21 epochs using the 60% threshold for pancreas detection, was fine-tuned. Organ inclusion metric was about 0.9, while the mask CD was 0.15 (SD: 0.17). Accuracy, precision, recall, and F1 score were 0.876, 0.874, 0.878, and 0.876, respectively (Table 7). The results showcased that, while there was a noticeable improvement between the base MiniGPT-v2 and MiniGPT-Pancreas for organ detection, the performances were aligned to fine-tuning GLM-4.1-9B-Base, a recently launched general-purpose MLLM. Remarkably, MiniGPT-Pancreas outperformed UMIT, a specific MLLM for the biomedical domain, pre-trained on M3D, a large dataset with 3D CTs [44]. When using a balanced dataset with the same number of slices with and without the pancreas, the IoU of MiniGPT-Pancreas improved, although it was computed only on the true positive cases. In this case, it achieved an accuracy of 0.864 on both the NIH and MSD datasets, in the classification of images with and without the pancreas. The second fine-tuning led to a boost in the metrics, exceeding MiniGPT-v2 by 0.40 for accuracy and precision, and about 0.50 for recall and F1. This highlighted the impact of the task identifier, which was VQA for the classification task, on model performances. Also in this task, MiniGPT-Pancreas outperformed both GLM-4.1V-9B-Base and UMIT.

**Table 7:** Tumor observation results. The best results are in bold. Accuracy, precision, recall, and F1 score are reported

Model	Acc	Prec	Rec	F1 Score
MiniGPT-v2	0.462	0.444	0.304	0.361
GLM-4.1V-9B-Base	0.682	0.617	0.875	0.724
UMIT^*^	0.500	0.500	1.000^**^	0.667
MiniGPT-Pancreas	**0.876**	**0.874**	**0.878**	**0.876**

^*^Only inference. ^**^The answer has always been “yes”

**Fig. 4:**
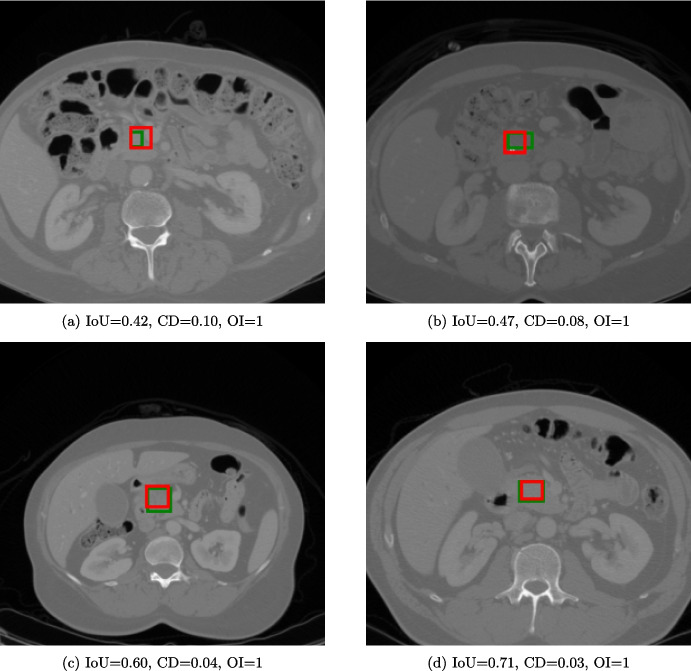
Four slices in the MSD dataset used for the pancreas tumor localization task. The green bounding box represents the GT, while the red represents the prediction

**Fig. 5:**
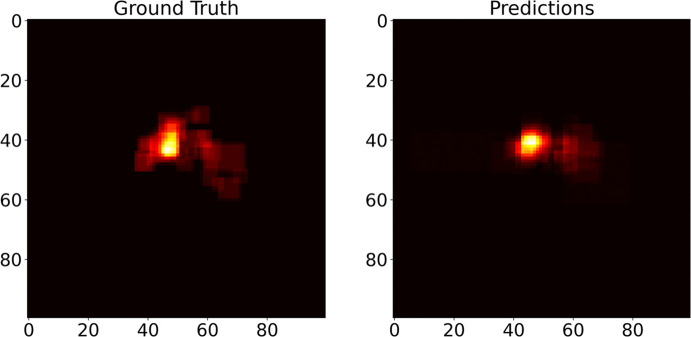
Heatmaps of the GTs and predicted bounding boxes for the pancreas tumor detection task

For tumor localization, we took the MiniGPT-Pancreas checkpoint from the initial detection task and fine-tuned it further using only the MSD dataset, chosen specifically because it contains detailed tumor annotations (see Section 3.6). As expected, localization performance was substantially lower than for whole-organ detection, yielding an average IoU of 0.280 across the test set. This drop reflects the small size and irregular shapes of pancreatic tumors compared to the organ itself. In this case, a more recent model like GLM-4.1V-9B-Base outperformed MiniGPT-Pancreas, thanks to its 3D convolutions in the image encoder. However, MiniGPT-Pancreas scored higher than UMIT (Table 8). Since IoU does not account for false positive cases, precision, recall, and F1 score were computed (threshold of 0.5), reporting 0.873, 0.747, and 0.805 on the MSD dataset. Although absolute IoU values remain modest, spatial accuracy metrics were stable, with a median centroid distance of 0.20 (SD: 0.16) and organ inclusion of 0.90 (SD: 0.30). Figure 4 showcases four representative cases: despite wide IoU variability, both CD and OI remain tightly clustered, underscoring the model’s consistent localization precision. To qualitatively support the CD and OI results, we generated normalized heatmaps of both ground-truth and predicted bounding-box centers (Fig. 5). These heatmaps demonstrated that, despite only moderate IoU scores, the model consistently located tumors with minimal centroid error and robust inclusion, underscoring its practical suitability for feasible clinical translation.

**Table 8:** Results on the tumor localization task. The best results are in bold

Model	IoU
MiniGPT-v2	< 0.001
GLM-4.1V-9B-Base	**0.486**
UMIT^*^	0.069
MiniGPT-Pancreas (Pancreas detection fine-tuning)	0.025
MiniGPT-Pancreas (Tumor observation fine-tuning)	0.072
MiniGPT-Pancreas (Tumor localization fine-tuning)	0.280
MiniGPT-Pancreas (balanced)^**^	0.249

^*^Only inference. ^**^Only for true positive cases

### Multi-Organ Detection

**Fig. 6:**
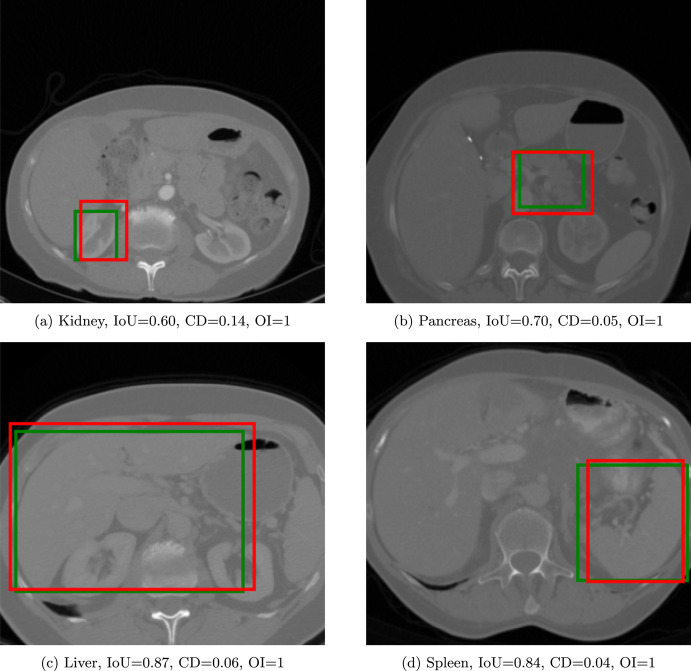
Examples of the multi-organ detection task using the AbdomenCT-1k dataset. The green bounding box represents the GT, while the red represents the prediction

Multi-organ detection ability was tested using the AbdomenCT-1K dataset, which provides annotations for the kidneys, liver, pancreas, and spleen. On average across the four organ types, IoU, CD, and OI were 0.68, 0.10, and 0.95, respectively. In this task, MiniGPT-Pancreas outperformed all models used as a benchmark. As expected, results confirmed that liver segmentation achieved the highest detection for all the tested MLLMs, followed in descending order by the kidneys, spleen, and pancreas (Tables 9 and 10). Notably, pancreas IoU on AbdomenCT-1K was roughly 8% lower than that observed on single-organ datasets such as NIH and MSD (Table 5), reflecting the increased challenge of distinguishing smaller structures in a multi-organ context. Figure 6 shows four illustrative cases: although IoU varies considerably across examples, CD and OI remain comparatively stable, underscoring consistent spatial precision despite fluctuating overlap metrics.

**Table 9:** Performance (IoU) for multi-organ detection on the AbdomenCT-1K dataset. Highest scores are in bold

	Kidney	Liver	Pancreas	Spleen
GLM-4.1V-9B-Base	0.64	0.75	0.43	0.69
UMIT^*^	0.07	0.48	0.03	0.02
MiniGPT-Pancreas	**0.71**	**0.84**	**0.50**	**0.70**

^*^Only inference

**Table 10:** Performance metrics (mean ± SD) for multi-organ detection on the AbdomenCT-1k dataset

Organ	IoU	CD	OI
Kidney	0.71(0.18)	0.06(0.09)	0.95(0.19)
Liver	0.84(0.12)	0.09(0.12)	0.99(0.07)
Pancreas	0.50(0.21)	0.16(0.12)	0.90(0.29)
Spleen	0.70(0.18)	0.11(0.23)	0.97(0.17)
Average	0.68	0.10	0.95

### Clinical Assessment

Ten expert general surgeons from international centers in Italy, Singapore, and the United Kingdom participated in the survey. They had a mean experience in surgery of 23.8 years, and executed a mean of 4660 surgical procedures as first operator. The results of the survey are reported in Table 11. The questions pertaining to the potential as a training tool achieved the highest score, with a mean of 4.67, followed by those on streamlining the clinical workflow (mean of 3.67) and addressing the clinical need (mean of 3.55).

**Table 11:** Results of the questionnaire administered to pancreatic surgery experts, expressed on a 5-point Likert scale with minimum=0, and maximum=5

	Mean score
Addressing the clinical need	
Can the system contribute to the early diagnosis of pancreatic cancer?	2.8
Can artificial intelligence help identify pancreatic lesions that radiologists might miss, reducing the risk of human error?	3.9
Can knowing the location of the pancreas, the location of the tumor, and the extent of the tumor help surgeons with preoperative planning?	4
Can the system improve communication between radiologists, surgeons, and oncologists?	3.5
Streamlining the clinical workflow	
Would integrating the system into existing hospital software be beneficial?	3.7
Can the system contribute to the standardization of diagnostic assessments?	4
Can the time needed for a clinical decision be reduced by using the system?	3.3
Potential as training platform	
Can the system be a valuable tool for training based on real cases?	4.4
Can the system be helpful as a verification tool for young doctors who may initially fail to detect pancreatic cancer, especially if it is small?	4.5
Can the system help in the training of young specialists (radiologists for diagnosis, or young surgeons to study pre-operative planning)?	4.5

## Discussion

The recent explosion of generative AI has fueled the development of increasingly powerful large language models (LLMs) that extend to multimodal applications. Yet applying MLLMs to pancreas imaging is uniquely challenging due to the organ’s small size, blurred boundaries, and high inter-patient variability in shape and position. In this proof-of-concept study, we present MiniGPT-Pancreas, the first multimodal LLM fine-tuned end-to-end for pancreas and tumor detection on CT slices, built atop MiniGPT-v2 and leveraging cascaded LoRA adapters.

### Key Findings

For pancreas detection and tumor observation, our findings have shown that MiniGPT-Pancreas outperformed the base MiniGPT-v2 model and the benchmark, represented by recent MLLMs, using the same fine-tuning strategy on GLV4.1-V-9B-Base, a general-purpose MLLM, or when directly evaluated at inference time on UMIT, an MLLM for several medical imaging tasks. In contrast, the performance of MiniGPT-Pancreas lagged behind that of GLM4.1-V-9B-Base for tumor detection. This may be due to the better capabilities of the 3D convolutions of the vision encoder of the latter than the 2D convolutions of the former to capture the very subtle features of the pancreatic tumors.

From a clinical point of view, MiniGPT-Pancreas was rated highly by a cohort of 10 expert surgeons in terms of potential as a training tool, especially for verification (4.5/5.0), and teaching purposes (4.5/5.0). Although overall, in each of the other domains of the questionnaire, MiniGPT-Pancreas scored lower, surgeons gave a relevant score on its capability to help in the surgical planning (4.0/5.0) and to reduce human error (3.9/5.0). In contrast, they did not foresee a role for MiniGPT-Pancreas to contribute to an early detection of pancreatic cancer (2.8/5.0) and to reduce the time to formulate a clinical decision. Anyway, the feedback gathered from these experienced surgeons will help determine the future development directions of MiniGPT-Pancreas within real-world clinical and educational settings.

### Comparison to Prior Works

Traditional CNN-based models for classification of pancreatic tumors yielded an accuracy of 0.832 when tested on the MSD and NIH pancreas datasets [46]. The task-specific cascaded fine-tuning of MiniGPT pancreas markedly enhanced accuracy to 0.88. In contrast, there is a lack of results on deep learning for the detection of the pancreas and its tumors. Most of the published studies on deep learning for pancreas imaging were actually on classification or segmentation. More specifically, deep learning architectures reached a Dice score of around 0.90 for pancreas segmentation on the NIH and MSD datasets. These performances degraded to around 0.60 for segmentation of pancreatic tumors [6]. Our findings highlighted that MiniGPT-Pancreas underperformed compared with deep learning for segmentation by an offset of approximately 0.30 in both detection tasks. The drop in the IoU can be attributed to the high sensitivity of this metric to small localization errors (quantifiable in a few pixels in some cases), especially when the GT bounding box is small, as in the case of pancreatic tumors. Moreover, our findings on both detection tasks represent a significant improvement compared to the base MiniGPT-v2 model. For the pancreas parenchyma detection, MiniGPT-Pancreas improved the IoU of MiniGPT-v2 by more than 11 and 12 times on the NIH and MSD datasets, respectively. For the tumor detection, the improvement exceeded two orders of magnitude. To a larger span, very recent works have begun to explore the application of LLMs in the management of pancreatic diseases. For example, [47] showed that ChatGPT’s responses to ten pancreatic cancer surgery questions were rated as equally comprehensible and empathetic as those from expert surgeons, with over 80% of patients expressing agreement on clarity and emotional resonance. In a clinical decision support setting, [48] evaluated 104 LLM-generated responses to NCCN-based scenarios on pancreatic ductal adenocarcinoma, finding that ChatGPT achieved 52% fully correct responses, outperforming Microsoft Copilot (33%), though 14% were still rated as inaccurate or misleading. Reference [49] demonstrated that a customized GPT designed for pancreatic cyst management achieved 87% concordance with expert recommendations across 60 clinical scenarios, with no significant difference in accuracy compared to specialists. In parallel, [50] reviewed AI applications in pancreatic cancer imaging and emphasized the need for improved detection accuracy across modalities, citing persistently low sensitivity and specificity in current segmentation and classification models, especially for small lesions. However, these prior studies were either text-only or based on general-purpose LLMs with limited ability to ground visual findings. In contrast, MiniGPT-Pancreas integrated unique vision and language modalities, tumor classification accuracy, and tumor localization. These results suggest that with targeted fine-tuning, compact multimodal LLMs can match or exceed the performance of prior LLM systems in both language and imaging tasks, bringing new capabilities to AI-assisted radiological workflows.

### Clinical and Technical Implications

Our results have highlighted that the fine-tuned model performed better for pancreas detection on datasets with only pancreas annotations than with multi-organ annotations. The observed difference of about 8% was due to the demanding task of detecting four organs simultaneously. MiniGPT-Pancreas may open up new scenarios to supplement clinicians for decision-making, and is flexible for several reasons. First, in contrast with other open-source MLLMs like LLaVA-Med and PeFoMed, it is not limited to the VQA, but it can be applied to other tasks, e.g., detection. Second, the results of multi-organ detection have shown that it can be used for the detection of other organs. Third, the bounding box generated by MiniGPT-Pancreas can be used as a prompt to guide foundation models like Segment Anything Model (SAM) for segmentation [51]. However, before the integration with models like SAM, the accuracy of organ detection must be improved. Fourth, from a technical perspective, we introduced two additional evaluation metrics, the centroid distance and organ/lesion inclusion, to complement standard IoU in assessing pancreas and tumor localization. These metrics are particularly valuable in the context of small and irregular anatomical structures, such as the pancreas and its tumors, where slight mismatches in bounding box alignment can drastically reduce IoU despite clinically acceptable localization. This dual evaluation provides a more robust assessment of localization performance. Technically, these metrics offer greater interpretability and stability, especially in low IoU but high-relevance cases, supporting the development of AI models that align more closely with radiological practice and downstream tasks such as attention-guided segmentation or triage.

### Limitations of the Study

This work has some limitations from both technical and clinical points of view. First, using the ViT 2D vision encoder means MiniGPT-Pancreas processes each CT slice independently, thus limiting its ability to exploit volumetric continuity, to maintain spatial coherence across adjacent slices, or to accumulate subtle lesion cues. It nonetheless offers significant practical advantages. Basically it leverages mature, large-scale pretrained 2D backbones (avoiding the data and annotation burdens of volumetric labeling), demands far less GPU memory and computation for faster training and inference, aligns naturally with slice-centric clinical workflows (e.g., PACS viewers), and provides a modular foundation that can later incorporate lightweight 3D aggregation (such as transformer-based fusion over slice embeddings) to recoup inter-slice context without retraining the core encoder from scratch. In summary, while a pure 2D encoder cannot capture volumetric context directly, it benefits from abundant pretrained backbones, lower annotation and compute requirements, and seamless integration into slice-centric clinical workflows. Future work could build on this solid 2D foundation by integrating lightweight 3D aggregation layers (e.g., transformer fusion over neighboring slice embeddings) to regain some volumetric coherence without forfeiting these practical advantages. Second, the radiological images used for training and testing were only CT slices of the abdominal organs containing the pancreas annotations. Therefore, we could not evaluate the performances on images from different modalities, e.g., MRI or ultrasound, or from different anatomical districts. In future work, we will enlarge the datasets with images from different modalities and organs. Third, the generalization ability of MiniGPT-Pancreas has so far been evaluated only on the pancreas organ using the AbdomenCT-1k multi-organ dataset, and not specifically on pancreatic cancer cases due to the current lack of publicly available, well-annotated datasets for this type of lesion. To ensure reliable performance in clinical settings, further validation on diverse, real-world datasets is essential, provided these datasets are curated in compliance with data protection regulations, such as the GDPR. Fourth, at present, MiniGPT-Pancreas supports only two core tasks: classification and detection. For broader clinical applicability, especially to assist physicians in complex diagnostic workflows, additional tasks, e.g., VQA, should be incorporated to enhance the model reasoning capabilities. Future development will focus on expanding MiniGPT-Pancreas to support a wider range of diagnostic tasks.

## Conclusion

Pancreatic tumors are very aggressive and are often diagnosed at an advanced stage. Recognizing the need to improve the accuracy at the early diagnosis stage, many AI models for pancreas imaging have been developed over the past decade. In this work, we presented MiniGPT-Pancreas, the first chatbot for the diagnosis of pancreatic tumors after fine-tuning MiniGPT-v2, a general-purpose MLLM. For this specific purpose, a cascaded fine-tuning was performed for the tasks of pancreas detection, tumor classification, and detection on the NIH and MSD publicly available datasets. The results have shown that the MiniGPT-Pancreas obtained high accuracy, precision, and recall on tumor classification, but it suffered in the detection of pancreatic tumors. Although the IoU on pancreas tumor detection is low, the model can detect tumors in an area close to the actual one, thus supporting young radiologists who may have initially missed the recognition of the cancer.

MiniGPT-Pancreas model have been made publicly available at: https://github.com/elianastasio/MiniGPT-Pancreas

## Figures and Tables

**Fig. 7:**
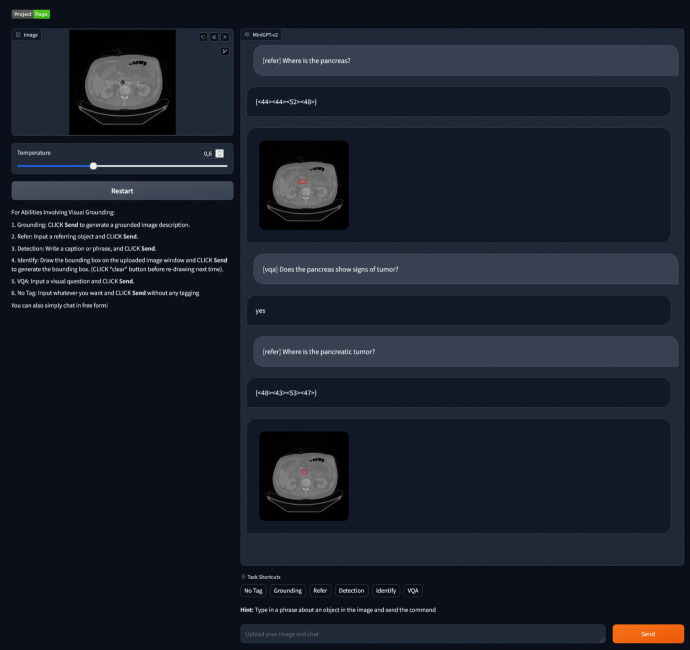
Example of one image-text pair, represented by a CT slice and a question, with corresponding generated output, represented by the bounding boxes coordinates, transformed into the actual bounding box superimposed to the initial CT image

**Fig. 8:**
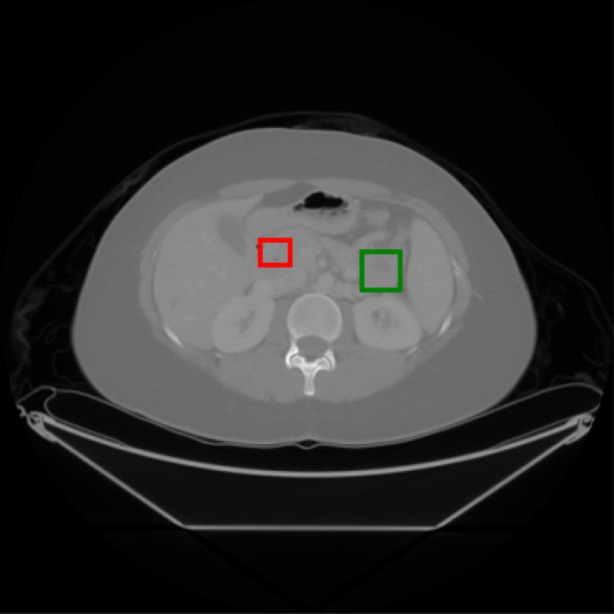
Example of one failure case where MiniGPT-Pancreas completely failed to detect pancreatic tumor on the MSD dataset

**Fig. 9:**
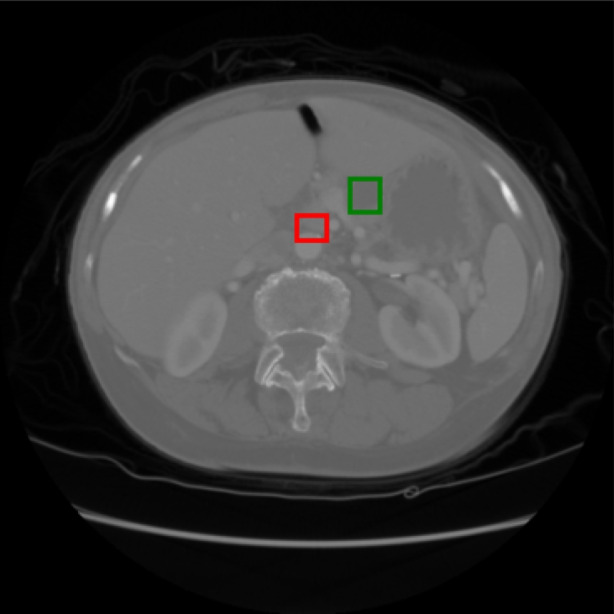
Example of one failure case where MiniGPT-Pancreas completely failed to detect pancreatic tumor on the MSD dataset

## Data Availability

No datasets were generated or analysed during the current study.

## Appendix A. JSON File Format

The following keys were used in the JSON file:

- **dataset**: Refers to the dataset of origin, e.g., the MSD dataset.
- **volume_name**: The name of the volume file, which contains the slice in question.
- **slice_id**: A unique identifier for the whole dataset.
- **slice_index**: The position of this slice within the entire volume.
- **slice_count**: The total number of slices in the volume.
- **pixels_pancreas**: The number of pixels labeled as pancreas in the slice. For the MSD dataset, this is the sum of pancreas pixels and tumor pixels.
- **pancreas_pixels_ratio**: The ratio of pancreas pixels to the maximum number of pancreas pixels found in any slice of the same volume. A value of 50%, for example, indicates that the current slice has half the pancreas pixels of the slice with the most pancreas pixels in that volume. This information is necessary for the adopted training approach, described in the next section.
- **max_pixels_pancreas**: The maximum number of pancreas pixels found in any slice of the same volume.
- **pixels_tumor**: The number of pixels identified as tumor in the slice. The information relative to the pancreatic tumor is, of course, present only for the MSD dataset.
- **tumor_pixels_ratio**: The ratio of tumor pixels to the maximum number of tumor pixels found in any slice of the same volume.
- **max_pixels_tumor**: The maximum number of tumor pixels found in any slice of the same volume.
- **bbox_pancreas**: The bounding box coordinates for the pancreas in this slice, provided as *(min*_*x*, *min*_*y*, *max*_*x*, *max*_*y**)*.
- **pancreas_bbox_ratio**: The ratio of the pancreas bounding box area to the maximum pancreas bounding box area found in any slice of the same volume.
- **max_bbox_pancreas**: The maximum area of the pancreas bounding box in any slice of the same volume.
- **bbox_tumor**: The bounding box coordinates for the tumor in this slice, provided as *(min*_*x*, *min*_*y*, *max*_*x*, *max*_*y**)*.
- **tumor_bbox_ratio**: The ratio of the tumor bounding box area to the maximum tumor bounding box area found in any slice of the same volume.
- **max_bbox_tumor**: The maximum area of the tumor bounding box in any slice of the same volume.
- **width**: The width of the image in pixels.
- **height**: The height of the image in pixels.

## Appenidx B. Normalized Center-Point Heatmap Computation

Let each bounding box $i$ in the test set have corner coordinates $\bigl (x_{\textrm{left}}^i,\,y_{\textrm{top}}^i\bigr )$ and $\bigl (x_{\textrm{right}}^i,\,y_{\textrm{bottom}}^i\bigr )$. We computed the heatmap in four steps:

1. **Centroid extraction:**
  $$x_c^i = \frac{x_{\textrm{left}}^i + x_{\textrm{right}}^i}{2}, \qquad y_c^i = \frac{y_{\textrm{top}}^i + y_{\textrm{bottom}}^i}{2}.$$
2. **Coordinate normalization:** Given image width $W$ and height $H$, it maps each centroid $(x_c^i,y_c^i)$ into the unit square as:
  $$u_i = \frac{x_c^i}{W}, \qquad v_i = \frac{y_c^i}{H}, \quad (u_i,v_i)\in [0,1]\times [0,1].$$
3. **Discrete heatmap construction:** To form a normalized 2D histogram over all $N$ centroids, a discrete (pixelized) heatmap *H*(*u*, *v*) was computed as:
  $$H(u,v) \;=\; \frac{1}{N}\sum _{i=1}^{N} \delta \bigl (u - u_i,\;v - v_i\bigr ), \quad \sum _{u,v} H(u,v) = 1,$$
  where $\delta$ is the discrete Kronecker delta on the chosen grid (e.g. 100 100 bins).
4. **Gaussian smoothing:** To make the visualization smoother and more interpretable, $H$ was convolved with a Gaussian kernel $G_{\sigma }$:
  $$H_{\textrm{smooth}}(u,v) = (H * G_{\sigma })(u,v) = \sum _{u',v'} H(u',v') \exp \!\Bigl (-\tfrac{(u - u')^2 + (v - v')^2}{2\sigma ^2}\Bigr ).$$
  Finally, $H_{\textrm{smooth}}$ was rescaled to $[0,1]$ by dividing by its maximum value.

## Appendix C. Examples

The graphical user interface (GUI) of MiniGPT-Pancreas with one example of image-text pair input and generated output, is showcased in Fig. 7. After uploading a CT slice along with a textual prompt (a question), MiniGPT-Pancreas generates the set of the bounding box coordinates, which are then used by the GUI to draw an actual bounding box, which is superimposed on the same input CT slice to produce the final result (Fig. 7).

Two failure cases, where MiniGPT-Pancreas completely failed to locate the pancreatic tumor in the MSD datasets, are depicted in Figs. 8 and 9.

## Footnotes

1 https://www.gradio.app/
